# Molecular Hydrogen as a Novel Antitumor Agent: Possible Mechanisms Underlying Gene Expression

**DOI:** 10.3390/ijms22168724

**Published:** 2021-08-13

**Authors:** Shin-ichi Hirano, Haru Yamamoto, Yusuke Ichikawa, Bunpei Sato, Yoshiyasu Takefuji, Fumitake Satoh

**Affiliations:** 1 Department of Research and Development, MiZ Company Limited, 2-19-15 Ofuna, Kamakura 247-0056, Kanagawa, Japan; y_ichikawa@e-miz.co.jp (Y.I.); b_sato@e-miz.co.jp (B.S.); info@e-miz.co.jp (F.S.); 2 MiZ Inc., 39899 Balentine Drive Suite 200, Newark, CA 94560, USA; haru.yamamoto@berkeley.edu; 3 Department of Molecular & Cell Biology, University of California, Berkeley, 3060 Valley Life Sciences Bldg #3140, Berkeley, CA 94720-3140, USA; 4 Faculty of Environment and Information Studies, Keio University (Professor Emeritus), 5322 Endo, Fujisawa 252-0882, Japan; takefuji@keio.jp; 5 Faculty of Data Science, Musashino University, 3-3-3 Ariake, Koto-Ku, Tokyo 134-8181, Japan

**Keywords:** molecular hydrogen, antitumor effect, gene expression, ROS, reactive oxygen species, DNA mutation, oxidative stress, antitumor agent, clinical application

## Abstract

While many antitumor drugs have yielded unsatisfactory therapeutic results, drugs are one of the most prevalent therapeutic measures for the treatment of cancer. The development of cancer largely results from mutations in nuclear DNA, as well as from those in mitochondrial DNA (mtDNA). Molecular hydrogen (H_2_), an inert molecule, can scavenge hydroxyl radicals (·OH), which are known to be the strongest oxidizing reactive oxygen species (ROS) in the body that causes these DNA mutations. It has been reported that H_2_ has no side effects, unlike conventional antitumor drugs, and that it is effective against many diseases caused by oxidative stress and chronic inflammation. Recently, there has been an increasing number of papers on the efficacy of H_2_ against cancer and its effects in mitigating the side effects of cancer treatment. In this review, we demonstrate the efficacy and safety of H_2_ as a novel antitumor agent and show that its mechanisms may not only involve the direct scavenging of ·OH, but also other indirect biological defense mechanisms via the regulation of gene expression.

## 1. Introduction

Tumors grow autonomously, against the control of the organism, and can be classified into malignant and benign tumors. Malignant tumors are characterized by autonomous growth, invasion, metastasis, and cachexia [1]. Benign tumors grow autonomously, but do not involve invasion, metastasis, and cachexia. Malignant tumors are broadly classified according to the organ or tissue in which they arise: carcinomas from epithelial cells, sarcomas from non-epithelial cells, hematopoietic malignancies from hematopoietic organs, and mesotheliomas from mesothelial cells [1].

According to a statistic from the International Agency for Research on Cancer (IARC), an external research organization of the World Health Organization (WHO), there were approximately 19 million cases of cancer and 10 million cancer deaths worldwide in 2020 [2]. Put differently, one third of all men and one quarter of all women develop some form of cancer in their lifetime, although there are slight differences between various countries and regions [2]. The three main treatment methods for cancer have traditionally been surgery, radiotherapy, and drug therapy. Cytotoxic anticancer drugs, such as platinum drugs, antitumor antibiotics, alkylating agents, antimetabolites and topoisomerase inhibitors, and molecular target drugs, such as tyrosine kinase inhibitors and antibody drugs, are often used in drug therapy [3,4]. In addition, nucleic acid drugs, such as antisense and small interfering RNA (siRNA), and immune checkpoint inhibitors, such as anti-programmed cell death 1(PD-1) antibody, anti-programmed cell death ligand 1 (PD-L1) antibody and anti-cytotoxic T-lymphocyte-associated protein 4 (CTLA-4) antibody, have also been used more recently [5,6]. However, none of these drugs have produced satisfactory therapeutic results [7]. With the development of modern medicine, diagnostic techniques and therapeutic measures for cancer have advanced, and the relative survival rate from cancer has improved year by year [2]. However, it still remains one of the major diseases that humans must overcome.

Molecular hydrogen (H_2_) is a flammable, colorless, odorless, and non-toxic gas. In 1975, Dole et al. first reported the potential medical applications of H_2_ [8]. They showed that hyperbaric treatment via the inhalation of 2.5% oxygen and 97.5% H_2_ gas significantly regressed squamous cell carcinoma in mice induced with ultraviolet (UV) irradiation. In 2007, Ohsawa et al. demonstrated that the inhalation of H_2_ gas (1–4%) significantly improved cerebral ischemia-reperfusion injury in a rat stroke model [9]. They found that the mechanisms of H_2_ against cerebral infarction involved the selective scavenging of hydroxyl radicals (·OH) and peroxynitrite (ONOO^−^), which are oxidative reactive oxygen species (ROS) and reactive nitrogen species (RNS), respectively [9]. This paper by Ohsawa et al. led to worldwide research on the medical applications of H_2_; however, Yanagihara et al. in 2005, two years before the reports by Ohsawa et al., showed that the oral administration of neutral electrolyzed water (1.6 ppm), containing H_2_ produced by electrolysis, in rats significantly ameliorated oxidative damages in the liver induced by chemical oxidants [10]. This work can be regarded as the pioneering study that motivates the potential medical applications of H_2_.

Research into the medical applications of H_2_ has made rapid progress. Unlike conventional medications, H_2_ has no side effects and is effective in treating many diseases caused by oxidative stress and chronic inflammation [11,12,13]. In a recent paper, we reported that H_2_ is the only molecule capable of mitochondrial translocation with ·OH scavenging ability [14]. H_2_ has clinical benefits for many diseases, including neurological diseases [15,16,17], cardiovascular diseases [18,19], respiratory diseases [20,21], diabetes [22], liver and metabolic syndrome [23,24]. Recently, many studies on H_2_ have reported its efficacy against cancer and its activity in improving the side effects of cancer treatments [25,26,27,28,29,30,31,32,33,34,35,36,37,38,39,40,41,42,43,44,45,46]. However, there have been no reviews that detail the potential of H_2_ as a novel antitumor agent and analyze its clinical applications with the possible mechanisms. On the other hand, with the recent developments in the molecular biology of cancer, the role of ROS in cancer has been elucidated in molecular genetic studies. In this paper, we review the efficacy of H_2_ as a novel antitumor agent and its underlying mechanisms from the viewpoint of gene expression. Moreover, we show the prospects of H_2_ as a novel antitumor agent in clinical applications.

## 2. Molecular Biology of Cancer

The human body is composed of about 37 trillion cells; approximately 1 trillion cells die and 1 trillion new cells are born each day [1]. These cells are created based on the genetic information in genomic DNA, but various factors can cause mutations in these genes, leading to the development of tumors in normal cells [1]. Even in a healthy person, thousands of cancerous cells are produced in the body every day. Genetic mutations can be induced by a variety of substances, including bacteria, viruses, parasites, chemical substances, ROS, ionizing irradiation, and UV light [1].

Not all mutated cells become cancerous cells, as these mutations develop into cancer through a multi-step process (multi-step carcinogenesis) [47]. The human body has a mechanism of apoptosis that stops cell division temporarily, checks for copy errors in its genes, tries to repair these errors, and kills itself off if it cannot be repaired [47]. There are also two types of oncogenes that both promote and suppress cancer cells. The p53 gene is activated when cells are subjected to oxidative stress [48]. The p53 gene acts as a transcriptional activator and plays an important role in cell cycle arrest, repair of genomic DNA, induction of apoptosis, inhibition of cancer cell metastasis, and angiogenesis [48]. For this reason, the p53 gene has been called the “guardian of the genome” [49]. However, if the normally active p53 gene is inactivated, not only is cell division unarrested, but abnormal cells do not undergo apoptosis and cancerous cells continue to grow after they have developed [48,49].

Mutations or deletions in the p53 gene are found in more than half of human cancers [50]. In addition, when the p53 gene is inactivated through methylation (an epigenetic change), normal cells turn into cancer cells [50]. However, if the body’s immune system is compromised due to ageing or poor lifestyle choices, the immune system may not be able to eliminate cancerous cells, and thus they may continue to proliferate. When cancerous cells grow to a visible size of 0.5 to 1 cm, they are referred to as cancer [1].

## 3. Redox Control in Cancer

**Figure 1:**
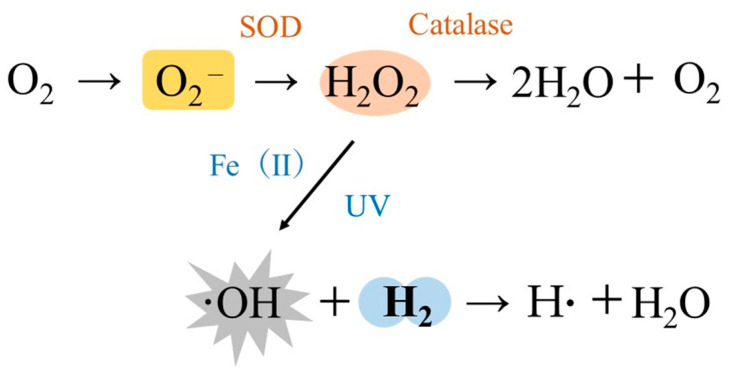
Selective action of molecular hydrogen (H_2_). Superoxide anion (O_2_^−^) is produced from oxygen that leaks from the mitochondrial electron transfer system. O_2_^−^ is further converted into hydrogen peroxide (H_2_O_2_). However, when water molecules are irradiated with ionizing radiation or H_2_O_2_ is irradiated with UV light, hydroxyl radicals (·OH) are produced. H_2_ can scavenge and detoxify the large amounts of ·OH produced in the mitochondria and other organelles by converting it into water molecules (·OH + H_2_ → H· + H_2_O).

An adult human consumes approximately 430 L of oxygen per day through respiration. However, 2–3% of ROS are produced by the body during this process [11,12,13]. Normally, the production of ROS and its scavenging systems are in balance; however, the excessive production of ROS due to smoking, alcohol consumption, air pollution, exposure to UV light, irradiation, strenuous exercise, and physical and psychological stress can induce oxidative stress that leads to a variety of harmful effects on the body [11,12,13]. There are four narrowly defined ROS in the human body: superoxide anions (O_2_^−^), hydrogen peroxide (H_2_O_2_), singlet oxygen (^1^O_2_), and ·OH [12]. The main site for the production of ROS in the cell is the mitochondria. Oxygen leaking from the mitochondrial electron transfer system is reduced to produce O_2_^−^, which is then converted to H_2_O_2_ by superoxide dismutase (SOD), and then to water by enzymes, such as catalase [11,12]. ·OH is produced when water molecules are irradiated with ionizing radiation or when H_2_O_2_ is irradiated with UV light. When there is an excess of iron in the vicinity of H_2_O_2_, a Fenton reaction occurs in which divalent iron ions react with H_2_O_2_ to form ·OH (Figure 1) [11,12]. Of these ROS, ·OH is about 100 times more potent than O_2_^−^ and is able to directly oxidize both nuclear DNA and mtDNA, but H_2_ can scavenge large amounts of ·OH produced in the mitochondria by converting it to water molecules [11,12,13].

Previous studies have shown that the main cause of cancer is abnormalities in genomic DNA [1]. Oxidative stress caused by ROS contributes to the genetic alteration of the carcinogenic process [1]. For example, many models of carcinogenesis show elevated level of 8-hydroxy-2′-deoxyguanidine (8-OHdG) in target organs early in the process [51]. 8-OHdG is a hydroxylated form of deoxyguanosine (dG), one of the bases of DNA. Since dG has the lowest redox potential amongst the four bases of DNA, it is susceptible to oxidation by ROS. 8-OHdG is one of more than 100 DNA-modifying bases that are produced in the highest amounts during oxidative stress [52]. 8-OHdG can be used as a biomarker to quantitatively reflect oxidative stress in vivo because it is chemically stable and is discharged into the blood and urine without undergoing secondary metabolism [53,54]. Oxidative stress has also been implicated in cell damage and carcinogenesis caused by ionizing irradiation. In a recent review, we demonstrated that the radioprotective effects of H_2_ may involve not only the direct scavenging of ·OH, but also antioxidant, anti-inflammatory, and anti-apoptotic effects via the regulation of gene expression [55].

The protein product of p53, a tumor suppressor gene, not only inhibits the development and progression of cancer, but also responds to various cellular stresses, such as hypoxia, viral infection, metabolic stress, endoplasmic reticulum stress, and oxidative stress. p53 also acts as a transcription factor, exhibiting effects, such as cell cycle arrest, DNA repair, angiogenesis suppression, senescence induction, and apoptosis induction. Since cellular stress is a cause of cancer, p53 suppresses the development and progression of cancer through these stress responses [48,56]. In particular, the mechanism by which oxidative stress causes the activation of the p53 gene is significant when considered as a mechanism by which the body maintains homeostasis. On the other hand, there are reports that p53 is involved in various phenomena in T-cell lymphoma, such as DNA repair, maintenance of mitochondrial proteins, and regulation of ribosome biogenesis, through functions different from the stress response, i.e., functions that do not involve transcription factors [57]. Methylation of the p53 gene is one of the main mechanisms for p53 gene inactivation and is a typical epigenetic change induced by DNA methyltransferases (DNMT) [58]. Currently, Azacitidine, a leading inhibitor of DNA methylation, is under clinical investigation for myelodysplastic syndromes (MDS) [59].

## 4. The Relationship between Cancer, Chronic Inflammation and Ageing

Inflammation is a tissue repair mechanism that maintains homeostasis in response to infections and tissue damages [60]. Acute inflammation is transient and reversible, whereas chronic inflammation is induced when inflammation is delayed by some disorder [60]. Current epidemiological evidence suggests that up to 25% of all cancers are associated with chronic infections and chronic inflammation [61]. Chronic inflammation and oxidative stress are closely linked, and mitochondria-specific radical scavengers can be used to control chronic inflammation. In a recent review, we showed that ·OH generated in the mitochondria induces oxidative stress in mitochondrial DNA (mtDNA), and that this oxidized mtDNA induces the activation of the nucleotide-binding and oligomerization domain-like receptor family pyrin domain containing 3 (NLRP3) inflammasome [60]. Since NLRP3 activation triggers a cascade of events leading to the release of pro-inflammatory cytokines, such as interleukin (IL)-1β and IL-18, we proposed that the mechanism by which H_2_ ameliorates chronic inflammatory diseases may involve, in part, the scavenging of ·OH produced in mitochondria [60]. On the other hand, the relationship between inflammation and cancer has been described as an exogenous pathway in which inflammation precedes cancer cells, and as an endogenous pathway in which changes in cancer cells, such as mutations in oncogenes, precede inflammation [62]. However, the relationship between cancer and inflammation is complex and requires further research.

Ageing can be divided into two categories: chronological ageing and cellular senescence. Cellular senescence is the cessation of the proliferation of normal cells due to their limited ability to divide. This was thought to be caused by the shortening of telomeres, the terminal structures of chromosomes [63]. However, recent studies have shown that cellular senescence is not only caused by telomere shortening, but also by oxidative stress and the activation of oncogenes, which can cause severe DNA damage [64]. Cellular senescence causes a phenomenon known as senescence-associated secretory phenotypes (SASP) that promote inflammation and carcinogenesis, via the secretion of inflammatory cytokines, chemokines, and extracellular matrix-degrading enzymes [65]. This suggests that the accumulation of cellular senescence may play a role in the development of chronic inflammation and cancer.

## 5. Antitumor Effects of H_2_

Reports on the antitumor effects of H_2_ can be broadly classified into literature investigating its efficacy in cells or animal models, and those investigating its clinical efficacy in human tumors (Table 1 and Table 2) [25,26,27,28,29,30,31,32,33,34,35,36,37,38,39,40,41,42,43,44,45,46]. The former can be categorized into investigations using cultured cancer cells, transplants of animal-specific tumor lines (allogeneic transplants), xenografts of human tumor lines in immunocompromised animals, or tumors induced in animals via exposure to UV or ionizing irradiation [25,26,27,28,29,30,31,32,33,34,35,36]. There are also reports of animals fed with a high-fat diet to induce nonalcoholic steatohepatitis (NASH) and evaluate its efficacy in the progression of liver cancer [37]. In addition, since the growth of cancer is accompanied by angiogenesis, the efficacy of cultured cells on angiogenesis has also been reported [38]. The following is a summary of literature reports that have investigated the efficacy of H_2_ on cancer.

### 5.1. Antitumor Effects in Cellular or Animal Models

#### 5.1.1. Antitumor Effects in Cellular Models

Saitoh et al. reported that neutral hydrogen-rich electrolytic water (NHE water, 0.5–1.1 ppm) reduced ·OH in ESR in a cell-free system [25]. They also investigated the effects of NHE water on human tongue carcinoma cells HSC-4 and normal human tongue epithelial-like cells DOK [25]. The results showed that NHE water decreased the colony formation rate and colony size of HSC-4 cells but did not inhibit those of DOK cells [25]. For human fibrosarcoma cells HT-1080, NHE water inhibited proliferation, cell degeneration, and invasion from reconstituted basement membranes. Furthermore, NHE water reduced intracellular total ROS in HSC-4 and HT-1080 cells [25].

Saitoh et al. reported in another paper the combined effects of platinum nano-colloid (Pt-nc) and hydrogen-dissolved water (HD water, 1.0–1.3 ppm) on HSC-4 and DOK cells [26]. The combination of HD water and Pt-nc enhanced the scavenging effect of ROS using the DPPH (diphenyl-picrylhydrazyl) radical scavenging method. In addition, the combination significantly reduced the colony formation rate and colony size of HSC-4 cells compared to HD water alone or Pt-nc alone, while it did not affect colonies of DOK cells. These results suggest that the enhanced antioxidant effects may be partly responsible for the mechanism of enhanced cell growth inhibition for the combination of HD water and Pt-nc [26].

Asada et al. also reported that the application of nanobubble H_2_ water (1.1–1.5 ppm) with platinum colloid in Ehrlich’s ascites tumor (EAT) cells resulted in the inhibition of ROS production and cell proliferation [27]. This combined effect was more pronounced than that of H_2_ water alone or platinum colloid alone, judging from the decrease in cell number, cell shrinkage and apoptosis [27]. Furthermore, this inhibitory effect on cell proliferation was enhanced in combination with hyperthermia at 42 °C, suggesting that nanobubble H_2_ water supplemented with platinum colloid may be an effective antitumor agent [27].

Saitoh et al. also found that the combined use of HD water (1.0–1.5 ppm) and Pt-nc in EAT cells had an inhibitory effect on cell proliferation, but this effect was significantly attenuated in the presence of catalase [28]. The combination also induced cell cycle arrest, with a decrease in the percentage of G1-phase cells and an increase in G2/M-phase cells. Furthermore, intracellular ROS levels were transiently and significantly increased immediately after H_2_ plus Pt-nc treatment, while the same did not occur after H_2_ or Pt-nc treatment alone. Based on these results, they suggested that the inhibitory effects of the combination of HD water and Pt-nc on cell proliferation may involve the transient and significant generation of H_2_O_2_ [28].

Kato et al. reported that electrolytic H_2_ water (0.6 ppm) combined with nanosized platinum-poly(N-vinyl-pyrrolidone) colloid (PVP-Pt) significantly enhanced the growth inhibition of human esophageal cancer-derived cells KYSE70 [29]. They demonstrated that the coexistence of 6-*O*-palmitoyl ascorbate (Asc6Palm) and PVP-Pt in electrolytic H_2_ water stabilized the esterified ascorbic acid, and that it increased the cellular uptake of PVP-Pt, resulting in the inhibition of cell proliferation [29].

On the other hand, Kagawa et al. investigated the effects of Pd-Ni hydrogen storage alloy (HSA) on cultured cancer cells (HeLa, H1299, SW and DLD1) and normal cells (MDCK, GP8, and NIH3T3) [30]. The results showed that HSA has no effect on normal cells, while it has a lethal effect on cancer cells near their surfaces. From these results, they inferred that the hydrogen radicals formed on the surface of HSA caused characteristic changes in cancer cells, and that the release of H_2_ may be responsible for its cancer cell-killing effects [30].

#### 5.1.2. Antitumor Effects in Animal Models

The antitumor effect of H_2_ was reported by Runtuwene et al. in in vivo and in vitro studies using colon 26, a mouse colorectal cancer-derived cell line [31]. H_2_ water (0.25 or 1.6 ppm) in combination with 5-fluorouracil (5-FU) was orally ingested by the mice. The combination increased the survival rate of mice compared to H_2_ water alone or 5-FU alone [31]. H_2_ water also showed a significant ROS scavenging effect, and the combination also improved the survival rate of cultured cells compared to each alone. Western blot analysis in cultured cells showed that the combination increased the expression of phosphorylated adenosine monophosphate activated protein kinase (p-AMPK), apoptosis-inducing factor (AIF), and caspase-3, suggesting that H_2_ water has antitumor properties through the activation of the apoptotic pathway [31].

Lung cancer is one of the most common and lethal malignancies in the world. Due to its high metastatic potential and drug resistance, lung cancer has a poor prognosis for patients. Wang et al. investigated the growth inhibitory effects of H_2_ gas (20–80%) on human lung cancer cell lines, A549 and H1975, and its mechanisms [32]. They also investigated the antitumor effects and mechanisms of the inhalation of H_2_ gas (60%) for 2 h per day in mice xenografted with A549 cells. The results showed that H_2_ gas inhibited cell proliferation and enhanced apoptosis in a concentration-dependent manner in vitro, and significantly inhibited tumor growth in vivo [32]. Furthermore, H_2_ gas suppressed the expression of ROS and increased the expression of SOD, IL-1β, IL-8, IL-13, and tumor necrosis factor-α (TNF-α) in lung tissue. Immunohistochemical staining of in vivo experiments also confirmed the repression of SMC3 expression by H_2_. Wang et al. suggested that H_2_ may inhibit lung cancer progression via the downregulation of the expression of SMC3, a regulator of chromosome condensation [32].

Zhao et al. synthesized palladium hydride (PdH) nanocrystals to develop tumor-targeted photoacoustic imaging (PAI)-guided hydrogen thermotherapy [33]. In order to evaluate the hydrogen heat therapy using PdH nanocrystals, two models were used: one with 4T1 breast cancer cells subcutaneously injected into the hind paw cavity of mice and the other with B16-F10 melanoma cells subcutaneously injected into the hind paw of nude mice. After implantation of each tumor, PdH nanocrystals were injected, followed by regular laser light irradiation to investigate the antitumor effects. The results showed that the combination of PdH nanocrystal injection and laser irradiation showed significant suppression of tumor volume and weight [33]. In addition, in cell culture experiments using HeLa human cervical cancer cells and HEK-293T human fetal kidney cells, the combination of PdH nanocrystals and laser irradiation had a strong inhibitory effect on HeLa cells but not HEK-293T cells. From these results, Zhao et al. reported that photothermal therapy with enhanced local production of H_2_ gas is effective in cancer treatment [33].

Glioblastoma (GBM) is the most common type of primary malignant brain tumors. Liu et al. investigated the antitumor effects of H_2_ gas (67%) on GMB using a rat orthotopic glioma model and a mouse subcutaneous xenograft model [34]. Inhalation of H_2_ gas for 1 h twice daily resulted in a significant growth inhibition of GMB tumors in both models and a significant prolongation of survival in the rat model. Immunohistochemistry and immunofluorescence staining for markers of stemness (CD133 and Nestin), proliferation (ki67) and differentiation (CD34) showed that the inhalation of H_2_ gas significantly reduced these markers in both models [34]. Inhalation of H_2_ gas also significantly increased the expression of the differentiation marker glial fibrillary acidic protein (GFAP). Similar results were obtained in an in vitro study using cultured cells. From these results, Liu et al. concluded that H_2_ inhibits the growth of GBM through the differentiation of glioma stem-like cells [34].

Yang et al. reported the possibility that pyroptosis via the terminal protein gasdermin D (GSDMD) pathway may be involved in the mechanism by which H_2_ exerts its antitumor effect on endometrial cancer [35]. The antitumor effects and mechanisms of H_2_ were investigated in experimental systems in which human endometrial cancer cell lines were cultured in vitro and xenografted into mice. The results showed that H_2_-rich water (1.2 ppm) increased ROS production, expression of pyroptosis-related proteins, number of TUNEL-positive cells, and expression of GSDMD in in vitro experiments using cultured cell lines [35]. In vivo experiments in mice also showed that the oral administration of H_2_-rich water reduced the volume and weight of tumors, and that the positive expressions of NLRP3, caspase-1, and GSDMD were significantly observed in tumor tissue sections from mice in the H_2_-rich water group. Yang et al. reported that the mechanism by which H_2_ inhibits the growth of endometrial cancer involves the pyrotrophic pathway via ROS/NLRP3/caspase-1/GSDMD [35].

#### 5.1.3. Antitumor Effects in UV or Ionizing Radiation Models

As described in the previous section, Dole et al. reported in *Science* the efficacy of the hyperbaric inhalation of 5% oxygen and 97.5% H_2_ gas on squamous cell carcinoma in mice induced by UV irradiation [8]. The mice in the H_2_ group demonstrated a significant inhibition of growth and regression of squamous cell carcinoma compared to control mice and mice in the group receiving hyperbaric inhalation of 5% oxygen and 97.5% helium gas. Dole et al. also suggested in this paper the possibility of an explosion of H_2_ gas [8]. At the same time, they suggested that the mechanism of the antitumor effects may involve the selective elimination of ·OH by H_2_. As described in the previous section, this paper is the first to report the potential medical applications of H_2_ gas as an antitumor agent [8].

Zhao et al. reported the protective effects of H_2_-rich saline (1.2 ppm) against thymic lymphoma induced by ionizing irradiation in BALB/c mice [36]. The control group received ionizing radiation for 4 weeks, and the H_2_ group received intraperitoneal administration of H_2_ rich saline 5 min before each ionizing radiation treatment. Compared with the control group, the H_2_ group showed a significant improvement in the survival of mice and a reduction in the incidence of lymphoma [36]. The H_2_ group also showed an inhibition in the production of ROS in peripheral blood mononuclear cells (PBMC). Furthermore, the H_2_ group showed a decrease in malondialdehyde (MDA) and an increase in SOD and glutathione (GSH) in plasma [36]. These results suggest a possible efficacy of H_2_ against cancer induced by ionizing irradiation.

#### 5.1.4. Inhibition of Carcinogenesis in the NASH Model

Oxidative stress has been strongly implicated in the pathogenesis from simple fatty livers to NASH, fibrosis, and hepatocarcinoma. Kawai et al. investigated the inhibitory effect of H_2_ on the progression to hepatocarcinogenesis in a STAM mouse model [37]. Two-day-old mice were treated with a single dose of streptozotocin to reduce insulin secretion and fed a high-fat diet from 4 weeks of age. H_2_-rich water (1.6 ppm) was administered to STAM mice for 8 weeks, and the number and size of liver tumors were examined. H_2_-rich water group showed a significant reduction in the number and size of tumors compared to the control group [37]. Immunohistochemical staining of proliferating cell nuclear antigen (PCNA) in the liver showed that the number of PCNA-positive nuclei in the H_2_-rich water group was significantly lower than in the control group [37]. These results suggest that H_2_-rich water may inhibit the progression of fatty liver to NASH, fibrosis, and hepatocarcinoma.

#### 5.1.5. Inhibitory Effects on Angiogenesis

Vascular endothelial growth factor (VEGF) is a key mediator of tumor angiogenesis. Tumor cells are exposed to higher oxidative stress than normal cells [38]. The intracellular redox state is closely related to the expression pattern of VEGF. Ye et al. reported the effects of electrolytic reduced water (ERW) obtained by water electrolysis on angiogenesis and its mechanism using cultured cells [38]. In co-culture experiments of human umbilical vein vascular endothelial cells (HUVEC) and human diploid embryonic lung fibroblasts (TIG-1), ERW significantly inhibited angiogenesis. ERW also reduced the release of H_2_O_2_ from A549 cells and decreased VEGF transcription and protein secretion. Furthermore, ERW inhibited the activation of extracellular signal-regulated kinase (ERK), which is involved in the regulation of VGEF expression. Based on these results, Ye et al. suggested that ERW may reduce VEGF gene transcription and protein secretion via the inactivation of ERK [38].

### 5.2. Antitumor Effects in Human Clinical Trials

Inactivation of the peroxisome proliferator-activated receptor gamma coactivator-1α (PGC-1α) results in reduced mitochondrial function, which leads to the exhaustion of CD8^+^ T cells and reduced antitumor immunity. As H_2_ has been reported to cause the activation of PGC-1α, Akagi et al. investigated the effects of H_2_ gas (67%) on immune function in a 3-month inhalation study in 55 patients with stage IV colorectal cancer [39]. The results demonstrated that H_2_ gas decreased exhausted terminal PD-1^+^ CD8^+^ T cells and increased activated PD-1^−^ CD8^+^ T cells in peripheral blood, significantly improving progression-free survival (PFS) and overall survival (OS) [39]. From these results, Akagi et al. suggested that the balance between PD-1^+^ CD8^+^ T and PD-1^−^ CD8^+^ T cells is important for cancer prognosis and that the recovery of exhausted CD8^+^ T cells may be involved in the mechanism by which H_2_ gas exerts its antitumor effects [39].

Since it was suggested that H_2_ gas may improve the prognosis of cancer patients by activating mitochondria, Akagi et al. investigated the effects of Nivolumab in combination with H_2_ gas, which is synergistic with mitochondrial activators [40]. Of 56 lung cancer patients treated with Nivolumab, 42 received inhaled H_2_ gas (67%) for up to 60 months. Results showed that patients treated with Nivolumab plus H_2_ gas inhalation had a significantly longer OS than those treated with Nivolumab alone [40]. Coenzyme Q10 (CoQ10) levels were also measured in these patients as a marker of mitochondrial function, and a multivariate analysis of CoQ10 levels and PD-1^+^Tim-3^+^ terminal CD8^+^ T cells (PDT^+^) was performed. Based on this analysis, Akagi et al. suggested that H_2_ gas may enhance the clinical efficacy of Nivolumab by increasing mitochondrial CoQ10 and decreasing PDT^+^ [40].

Chen et al. treated 82 cancer patients with inhaled H_2_ gas (67%) for at least 3 h per day for at least 3 months [41]. 34% of patients were treated with H_2_ gas inhalation alone, but the remaining 66% of patients used several anticancer drugs in small doses as adjuncts to the H_2_ gas inhalation. After 4 weeks, 41.5% of patients had an improvement in quality of life (QOL), including improvements to fatigue, insomnia, appetite, and pain. Complete and partial remissions occurred between 21 and 80 days (median 55 days) after H_2_ gas inhalation, with an overall disease control rate of 57.5% [41]. The disease control rate was significantly higher in stage III patients than in stage IV patients (83.0% and 47.7%, respectively), with the lowest disease control rate in patients with pancreatic cancer. From these results, Chen et al. concluded that H_2_ gas inhalation is a treatment that can improve the QOL of cancer patients and inhibit cancer progression [41].

Chen et al. also reported the results of a case study of H_2_ gas (67%) inhalation therapy for patients with metastatic gallbladder cancer with a primary site in the liver [42,43]. During one month of H_2_ gas inhalation therapy (2–6 h/day), the gallbladder and liver cancers continued to progress and were complicated by bowel obstruction. However, in parallel with the H_2_ gas therapy, symptomatic treatment of the bowel obstruction gradually improved and the metastases in the abdominal cavity gradually decreased. Furthermore, the patient’s anemia improved, and their lymphocyte and tumor markers returned to normal levels [42,43]. They reported that the patient was able to resume a normal life two and a half months after H_2_ gas inhalation and is still alive after more than 10 months [42,43].

Lung cancer is a very metastatic cancer, able to spread to the opposite lung, bone, and brain. Therefore, Chen et al. also reported the results of a case study of a patient with a brain tumor that had multiple metastases from lung cancer [44]. The patient was treated with standard therapy that had no effects, and metastases transferred from the lungs to the brain, bones, adrenal glands, and liver. After 4 months of monotherapy with H_2_ gas (67%) for 2–6 h per day, the size of several brain tumors was significantly reduced, as was the amount of spinal fluid from hydrocephalus associated with the brain tumors. They reported that after one year, all brain tumors had disappeared and the increase in the size of the lung and liver cancers had been controlled [44].

Chen et al. divided 58 patients with advanced non-small cell lung cancer into five groups: control (10 patients), H_2_ alone (10 patients), H_2_ plus chemotherapy (10 patients), H_2_ plus targeted therapy (18 patients), and H_2_ plus immunotherapy (10 patients) [45]. All groups excluding the control group received H_2_ gas inhalation (67%) for 4–5 h a day for 5 months. During the first five months of treatment, the prevalence of symptoms gradually increased in the control group, while it decreased in the four treatment groups. The 16 months of follow-up demonstrated that progression-free survival rates in the H_2_ alone, H_2_ plus chemotherapy, H_2_ plus targeted therapy, and H_2_ plus immunotherapy groups were significantly higher than in the control group [45]. Most side effects of drugs were reduced or eliminated in the combination therapy group. From these results, Chen et al. concluded that inhaled H_2_ gas can be used to reduce tumor progression and alleviate drug side effects in patients [45].

Chen et al. also enrolled 20 non-small cell lung cancer patients and assessed the immune senescence of peripheral blood lymphocyte subsets, including T cells, natural killer T cells, and gamma-delta (γδ) T cells [46]. During the waiting period for tests on treatment, the patients were treated with 4 h of daily H_2_ gas (67%) inhalation for 2 weeks, and none of the patients received standard treatment. After 2 weeks of H_2_ treatment, exhausted and senescent cytotoxic T cells decreased to within normal limits, and killer Vδ1 cells increased. Abnormally reduced indicators included functional helper T cells and cytotoxic T cells, Th1, total natural killer T cells, natural killer, and Vδ2 cells. Based on these results, they reported that 2 weeks of H_2_ gas inhalation could significantly reverse adaptive and innate immune senescence in patients with advanced non-small cell lung cancer [46].

## 6. Possible Mechanisms of the Antitumor Effects of H_2_

**Figure 2:**
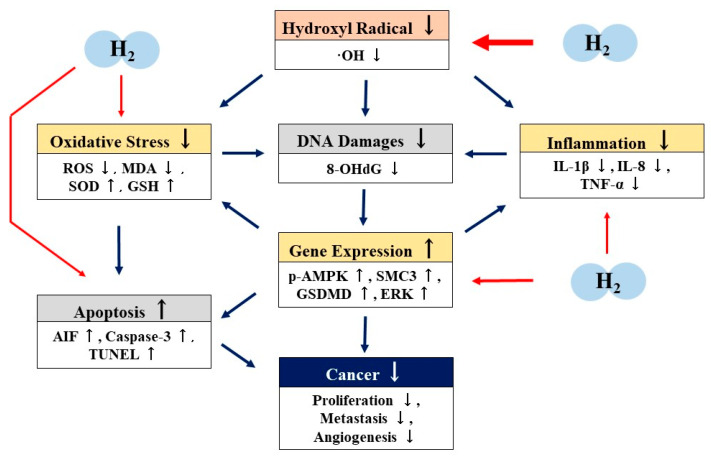
Possible mechanism of the antitumor effects of molecular hydrogen (H_2_.) H_2_ scavenges hydroxyl radicals (·OH) directly. H_2_ also exhibits antioxidant, anti-inflammatory, and apoptotic effects through the regulation of gene expression indirectly. Through these direct and indirect actions, H_2_ may exhibit antitumor effects. H_2_: molecular hydrogen; ·OH: hydroxyl radicals; ROS: reactive oxygen species; MDA: malondialdehyde; SOD: superoxide dismutase; GSH: glutathione; 8-OHdG: 8-hydroxy-2′-deoxyguanidine; p-AMPK: phosphorylated adenosine monophosphate activated protein kinase; AIF: apoptosis-inducing factor; TUNEL: TdT-mediated digoxygenin (biotin)-dUTP nick end labeling; SDMD: gasdermin D; ERK: extracellular signal-regulated kinase; IL-1β: interleukin-1β; IL-8: iterleukin-8; TNFα: tumor necrosis factor-α.

Many cellular, animal, and clinical studies have shown that H_2_ has an excellent anti-tumor effect [25,26,27,28,29,30,31,32,33,34,35,36,37,38,39,40,41,42,43,44,45,46]. However, there are few reports on the mechanisms of the antitumor effects of H_2_. Based on these reports, we propose that the mechanisms of the antitumor effects of H_2_ may involve not only the direct scavenging of ·OH, but also indirect effects such as antioxidation, anti-inflammation, apoptosis, or pyroptosis via the regulation of gene expression (Figure 2).

The inhibition of ·OH or total ROS production in the antitumor effects of H_2_ have been reported in many papers [25,26,27,31,32,33,36]. Wang et al. reported increased SOD activity, suppressed expression of IL-1β, IL-8, TNF-α, and enhanced apoptosis in the antitumor effects of H_2_ [32]. Zhao et al. reported that H_2_ increases SOD and GSH activities while decreasing MDA [36], and Yang et al. reported that H_2_ enhances pyroptosis [35]. On the other hand, in terms of gene expression related to these antitumor effects, Runtuwene et al. reported the increased expression of p-AMPK, AIF, and caspase-3 in the antitumor effects of H_2_ by Western blot analysis [31]. Wang et al. reported the repression of SMC3 expression by H_2_ in Western blot analysis and immunohistochemical analysis [32], and Yang et al. reported the increased expression of GSDMD by H_2_ in experiments examining mRNA expression [35]. Ye et al. reported inhibition of ERK activation, which is involved in the regulation of VGEF expression, in an experimental system for angiogenesis using cultured cells [38]. Moreover, Kawai et al. demonstrated that drinking H_2_ water significantly reduced plasma levels of 8-OHdG in another experimental NASH model induced by a high-fat diet [37].

The mechanism of the antitumor effects of H_2_ gas in human clinical trials have been reported by Akagi et al. [39,40] and Chen et al. [42,43]. In cancer patients, mitochondrial function is reduced due to the inactivation of PGC-1α. However, they reported that the inhalation of H_2_ gas activated PGC-1α and antitumor immunity by increasing PD-1^−^ CD8^+^ T cells. Similarly, Chen et al. reported that the inhalation of H_2_ gas improved adaptive and innate immune senescence in cancer patients [46]. Although only a decrease in antitumor immunity in cancer patients is reported, this decrease may be attributed to impaired mitochondrial function, one of these factors being oxidative stress. It is well known that ROS-induced oxidative stress causes mtDNA mutations and a decrease in mtDNA levels, ultimately leading to mitochondrial dysfunction, including dysfunction of the respiratory chain. Kamimura et al. reported that the enhanced expression of the PGC-1α gene is involved in the mechanism by which H_2_ water administration in obese mouse models enhances fat metabolism [66]. Although further detailed mechanistic studies are needed, it is possible that the activation of antitumor immunity by H_2_ gas involves not only the direct scavenging of ·OH, but also biological defense mechanisms through the regulation of gene expression (Figure 2).

The reaction rate of H_2_ with ·OH in aqueous solution is reported to be much slower than those with DNA, amino acids, sugars, and GSH [67]. However, the reaction rate between ·OH and H_2_ in aqueous solution does not apply to the high concentration of biological components in the cytoplasm and nucleus. Since H_2_ is the smallest molecule and has a high diffusion rate, the diffusion rate of H_2_ into the mitochondria and nucleus may contribute; H_2_ can diffuse through biological membranes to reach the mitochondria, the main site of ·OH production, and repair mutations in mtDNA [11,12]. However, since ·OH has a very short half-life [68], it is unlikely that the ·OH produced in the mitochondria will migrate into the nucleus and react with nuclear DNA. Rather, there is a possibility that ·OH generated from water molecules in the nucleus by irradiation excitation will react with DNA. In addition, H_2_O_2_ in the nucleus may produce ·OH via UV irradiation or the enhancement of the Fenton reaction, and this ·OH may also cause mutation of nuclear DNA. Therefore, H_2_ can efficiently reduce ·OH generated not only in mitochondria but also in the nucleus, thereby protecting nuclear DNA from mutations.

## 7. Prospects of H_2_ as an Antitumor Agent

Drug therapy (chemotherapy) is one of the most important treatment options for cancer. Chemotherapy with antitumor agents may be used as a treatment alone or in combination with pre- or post-surgery radiotherapy. Alkylating agents [69], metabolic antagonists [70], antitumor antibiotics [71], and plant alkaloids [72] have long been used clinically as antitumor agents, but these agents damage not only cancer cells but also normal cells. While the mechanisms of the side effects of these drugs have been investigated, and their derivatives and new antitumor agents with fewer side effects have also been developed [73,74,75,76], the problem with the side effects has not been satisfactorily solved. More recently, molecularly targeted agents have been developed, and nucleic acid drugs and immune checkpoint inhibitors have also been introduced [5,6]. However, despite the expansion of therapeutic options, concerns about efficacy and safety remain, and the emergence of a novel antitumor agent with higher efficacy and reliable safety is desirable.

ROS-induced oxidative stress can cause mutations in normal cells and promote their transformation into cancer cells [77,78]. ROS also promote the stabilization of factors that promote cancer initiation and progression [77,78]. Therefore, a method to eliminate ROS in the body and reduce oxidative stress by antioxidants is promising for the prevention and treatment of cancer. A large clinical trial with vitamin E supplementation was therefore conducted [79]. However, the results were contrary to expectations: there was a significant increase in the incidence of prostate cancer in patients who received vitamin E [79,80]. Studies in mouse models of cancer also confirmed the accelerated effects of supplementation with N-acetyl cysteine and vitamin E on cancer development [81]. The mechanisms by which these antioxidants promote cancer growth has been reported by DeNicola et al. and Schafer et al. [82,83]. From these results, it is suggested that the effects of antioxidants on cancer are double-sided, and that depending on the conditions, they may either inhibit or promote cancer development.

The most abundant ROS in the body is O_2_^−^, followed by H_2_O_2_, although ·OH has the strongest oxidizing power. Most antioxidants, with the exception of H_2_, are not selective for ·OH, yet they are able to scavenge O_2_^−^ and H_2_O_2_, which play important roles in the body such as infection control and signal transduction [11,12]. H_2_, on the other hand, scavenges only ·OH, the most abundant form of ROS in mitochondria, and has no direct scavenging effects on other ROS, such as O_2_^−^ or H_2_O_2_ [11,12,13]. The small molecular size of H_2_ allows it to rapidly cross cell membranes and diffuse into the cytoplasm [11,12,13,53,57]. It then reaches the nucleus and mitochondria of the cell within a short span of time and protects both nuclear DNA and mtDNA [11,12,13]. However, other antioxidants have a much lower permeability into the cell than H_2_ [11,12]. This difference in selectivity for ROS and intracellular kinetics may be responsible for the fact that H_2_ shows antitumor effects, while other antioxidants have both antitumor and pro-carcinogenic effects depending on the conditions.

Akagi et al. reported that the mechanisms of the antitumor effects of H_2_ gas involves the decrease in PD-1^+^ CD8^+^ T cells and the increase in PD-1^−^ CD8^+^ T cells [39,40]. Based on these results, it can be assumed that H_2_ also exhibits pro-inflammatory activity. Contradictory effects have been found in many studies, as H_2_ can show pro-inflammatory activity and immuno-potentiating effects, while it also possesses anti-inflammatory and immunosuppressive effects [39,40,84,85,86]. This may seem contradictory, but H_2_ can be considered to be double-sided depending on the experimental conditions. In other words, H_2_ shows both pro-inflammatory activity and anti-inflammatory effects on inflammatory systems, as well as potentiating and suppressive effects (immunomodulating activity) on immune systems. This suggests that the effects of H_2_ on inflammation and immunity always work as a potentiator or inhibitor to maintain the homeostasis of the body.

In this study of the antitumor effects of H_2_, various methods of application were used to ingest H_2,_ including the inhalation of H_2_ gas, ingestion of H_2_-rich water, and intraperitoneal injection of H_2_-rich saline. Each of these administration methods has its own characteristics, but we believe that the inhalation of H_2_ gas can supply the highest amount of H_2_ in a time-dependent manner. This is explained by the fact that the maximum blood and tissue concentration (Cmax) of H_2_ gas inhalation is lower than that of other routes of administration, while the area under the curve (AUC) is extremely high [87,88]. On the other hand, we can divide these studies into two groups: those that examined the effect of H_2_ alone and those that examined the effect of H_2_ in combination with other drugs. For example, the combination studies include the combination of H_2_ dissolved water with platinum colloid [26,28], the combination of H_2_ water with 5-Fu [31], and the combination of H_2_ gas with Nivolumab [40]. Since all of these studies examining the combined effects showed greater effects than the H_2_ alone, it can be assumed that the combination of H_2_ and these agents compensates for the shortcomings of the effects of H_2_ alone and shows additive or synergistic effects.

The history of research for H_2_ is extremely old, since the antitumor effect of H_2_ was first reported by Dole et al. in 1975 [8]. However, it is only recently that H_2_ has been reported to show antitumor activity, particularly in in vivo and human clinical studies, and the antitumor properties of H_2_ have not been highlighted. As outlined in this review, H_2_ has demonstrated excellent antitumor activity in various cellular models, animal models and human clinical trials [25,26,27,28,29,30,31,32,33,34,35,36,37,38,39,40,41,42,43,44,45,46]. More than 1000 papers have been published on the medical applications of H_2_, including more than 80 reports of human clinical trials [55]. These papers confirm that H_2_ is highly effective in a variety of diseases and that there are no safety issues. In the field of drug therapy for cancer, new drugs, such as nucleic acid drugs [5] and immune checkpoint inhibitors [6], are being developed, while there are many problems with their efficacy and safety. Therefore, the clinical application of H_2_ as a novel antitumor agent could potentially carve a new path in the field of cancer treatment.

## 8. Conclusions

H_2_ has been reported to have excellent efficacy and safety in many diseases [15,16,17,18,19,20,21,22,23,24]. Recently, several research studies on the efficacy of H_2_ against cancer and the improvement of cancer treatment side effects have been reported [25,26,27,28,29,30,31,32,33,34,35,36,37,38,39,40,41,42,43,44,45,46]. However, there have been no reviews that outline the potential of H_2_ as a novel antitumor agent with clinical applications and analyses of its mechanisms. In this paper, we reviewed the efficacy and mechanisms of H_2_ as a novel antitumor agent from the viewpoint of gene expression. H_2_ shows excellent antitumor efficacy in cellular models, animal models, and human clinical trials [25,26,27,28,29,30,31,32,33,34,35,36,37,38,39,40,41,42,43,44,45,46], and the mechanism of efficacy may involve not only the direct scavenging of ·OH by H_2_, but also the indirect biological defense mechanisms via the regulation of gene expression. H_2_ has shown excellent efficacy and safety as an antitumor agent and its clinical application may provide a new therapeutic strategy against cancer.

## Figures and Tables

**Table 1:** Summary of antitumor effects of molecular hydrogen (H_2_) in cellular models and animal models.

Experimental System	Cancer Types	Effects of Molecular Hydrogen (H_2_)	Ref. No.
Cultured cells	Human tongue cancer and Human fibrosarcoma	H_2_-rich electrolyzed water showed inhibitory effects on ROS production and cell proliferation.	[25]
	Human tongue cancer and Human fibrosarcoma	H_2_-dissolved water, in combination with platinum nano-colloid, showed enhanced suppression of ROS production and cell proliferation.	[26]
	Mouse Ehrlich’s ascites tumor	Nanobubble H_2_ water with platinum nano-colloid demonstrated inhibitory effects on the production of ROS and cell proliferation.	[27]
	Mouse Ehrlich’s ascites tumor	The combination of H_2_-dissolved water and platinum nano-colloid showed an inhibitory effect on cell proliferation, and the effect involved the transient generation of hydrogen peroxide.	[28]
	Human esophageal cancer	The combination of electrolytic H_2_ water and PVP-Pt colloid demonstrated enhanced inhibition of cell proliferation.	[29]
	Human cervical cancer, Human soft tissue tumor, etc.	Hydrogen radicals and H_2_ produced from Pd-Ni hydrogen storage alloys showed a killing effect on four types of tumor cells.	[30]
Experimental animals (Transplant models)	Mouse colon cancer	The combination of H_2_ water and 5-fluorouracil showed antitumor effects via activation of the apoptotic pathway.	[31]
	Human lung cancer	H_2_ gas showed antitumor effects through down-regulation of the expression of SMC3, a regulator of chromosome condensation.	[32]
	Mouse breast cancer and Human melanoma	The combination of palladium hydride and laser light irradiation was effective in suppressing tumor volume and tumor weight.	[33]
	Rat glioma and Human glioma	H_2_ gas inhibited the growth of glioblastoma through differentiation of glioma stem-like cells.	[34]
	Human endometrial cancer	H_2_-rich water showed antitumor effects by activating the ROS/NLRP3/caspase-1/GSDMD-mediated pyrotrophic pathway.	[35]
Experimental animals (Induced models)	UV-induced skin cancer	Hyperbaric treatment with H_2_ gas inhibited the growth and regression of skin cancer in mice induced by UV.	[8]
	Ionizing radiation-induced thymic lymphoma	H_2_-rich saline suppressed the development of thymic lymphoma in mice induced by ionizing radiation.	[36]
	High-fat diet-induced liver cancer	H_2_-rich water showed carcinogenic effects in an experimental system that progressed from NASH to fibrosis and liver cancer.	[37]
Angiogenesis	Co-culture experiment of 2 cell lines	Electrolyzed reduced H_2_ water inhibited lumen formation via suppression of VEGF expression in cultured cells.	[38]

H_2_: molecular hydrogen; ROS: reactive oxygen species; PVP-Pt: platinum-poly(N-vinyl-pyrrolidone); NLRP3: nucleotide-binding and oligomerization domain-like receptor family pyrin domain containing 3; GSDMD: gasdermin D; UV: ultraviolet; NASH: nonalcoholic steatohepatitis; VEGF: vascular endothelial growth factor.

**Table 2:** Summary of antitumor effects of molecular hydrogen (H_2_) in human clinical trials.

Cancer Types	No. of Patients	Administration Periods	Effects of Molecular Hydrogen (H_2_)	Ref. No.
Colorectal cancer	55	3-months	Inhalation of H_2_ gas prolonged progression-free survival and overall survival in patients with stage IV, and the mechanism involved the recovery of CD8^+^ T cells.	[39]
Lung cancer	42	60-months	The combination of H_2_ gas and Nivolumab showed prolonged overall survival, and the mechanism involved increased coenzyme Q10.	[40]
Lung cancer, liver cancer, pancreatic cancer, etc.	82	At least 3-months	Inhalation of H_2_ gas in patients with stage III or IV resulted in complete (CR) and partial remission (PR), with a disease control rate of 57.5%.	[41]
Gallbladder cancer	1	10-months	A case study of a patient with gallbladder cancer whose primary tumor was in the liver showed that inhalation of H_2_ gas had a tumor-reducing effect.	[42,43]
Brain tumor	1	4-months	A case study of a patient with a brain tumor whose primary tumor was lung cancer showed that inhalation of H_2_ gas had a tumor-reducing effect.	[44]
Lung cancer	48	5-months	Treatment with H_2_ gas inhalation alone (10 patients) or in combination with anticancer drugs (38 patients) prolonged progression-free survival in lung cancer patients.	[45]
Lung cancer	20	2-weeks	Inhalation of H_2_ gas improved adaptation and innate immune senescence in lung cancer patients.	[46]

## Data Availability

The data presented in this study are available on request from the corresponding author.

## References

[1] National Cancer Institute What Is Cancer?. https://www.cancer.gov/about-cancer/understanding/what-is-cancer.

[2] Sung H., Ferlay J., Siegel R.L., Laversanne M., Soerjomataram I., Jemal A., Bray F. (2021). Global cancer statics 2020: GLOBOCAN estimates of incidence and mortality worldwide for 36 cancers in 185 countries. CA Cancer J. Clin..

[3] Kindler H.L. (2000). Malignant pleural mesothelioma. Curr. Treat. Options Oncol..

[4] Lee Y.T., Tan Y.J., Oon C.E. (2018). Molecular targeted therapy: Treating cancer with specificity. Eur. J. Pharmacol..

[5] Opalinska J.B., Gewirtz A.M. (2002). Nucleic-acid therapeutics: Basic principals and recent applications. Nat. Rev. Drug Discov..

[6] Xu C., Chen Y.P., Du X.J., Liu J.Q., Huang C.L., Chen L., Zhou G.Q., Li W.F., Mao Y.P., Hsu C. (2018). Comparative safety of immune checkpoint inhibitors in cancer: Systematic review and network meta-analysis. BMJ.

[7] Vasan N., Baselga J., Hyman D.M. (2019). A view on drug resistance in cancer. Nature.

[8] Dole M., Wilson F.R., Fife W.P. (1975). Hyperbaric hydrogen therapy: A possible treatment for cancer. Science.

[9] Ohsawa I., Ishikawa M., Takahashi K., Watanabe M., Nishimaki K., Yamagata K., Katsura K.I., Katayama Y., Asoh S., Ohta S. (2007). Hydrogen acts as a therapeutic antioxidant by selectively reducing cytotoxic oxygen radicals. Nat. Med..

[10] Yanagihara T., Arai K., Miyamae K., Sato B., Shudo T., Yamada M., Aoyama M. (2005). Electrolyzed hydrogen-saturated water for drinking use elicits an antioxidative effect; a feeding test with rats. Biosci. Biotrechnol. Biochem..

[11] Ohta S. (2014). Molecular hydrogen as a preventive and therapeutic medical gas: Initiation, development and potential of hydrogen medicine. Pharmacol. Ther..

[12] Ohta S. (2015). Molecular hydrogen as a novel antioxidant: Overview of the advantages of hydrogen for medical applications. Methods Enzymol..

[13] Hirano S.-i., Ichikawa Y., Sato B., Satoh F., Takefuji Y. (2020). Hydrogen is promising for medical applications. Clean Technol..

[14] Hirano S.-i., Ichikawa Y., Kurokawa R., Takefuji Y., Satoh F. (2020). A “philosophical molecule,” hydrogen may overcome senescence and intractable diseases. Med. Gas Res..

[15] Ono H., Nishijima Y., Adachi N., Tachibana S., Chitoku S., Mukaihara S., Sakamoto M., Kudo Y., Nakazawa J., Kaneko K. (2011). Improved brain MRI indices in the acute brain stem infarct sites treated with hydroxyl radical scavengers, Edaravone and hydrogen, as compared to Edaravone alone. A non-controlled study. Med. Gas Res..

[16] Yoritaka A., Takanashi M., Hirayama M., Nakahara T., Ohta S., Hattori N. (2013). Pilot study of H2 therapy in Parkinson’s disease. A randomized double-blind placebo-controlled trial. Mov. Disord..

[17] Ono H., Nishijima Y., Ohta S., Sakamoto M., Kinone K., Horikoshi T., Tamaki M., Takeshita H., Futatuki T., Ohishi W. (2017). Hydrogen gas inhalation treatment in acute cerebral infarction: A randomized controlled clinical study on safety and neuroprotection. J. Stroke Cerebrovasc..

[18] Katsumata Y., Sano F., Abe T., Tamura T., Fujisawa T., Shiraishi Y., Khosaka S., Ueda I., Honmma K., Suzuki M. (2017). The effects of hydrogen gas inhalation on adverse left ventricular remodeling after percutaneous coronary intervention for ST-elevated myocardial infraction. First pilot study in humans. Circ. J..

[19] Tamura T., Hayashida K., Sano M., Suzuki M., Shibusawa T., Yoshizawa J., Kabayashi Y., Suzuki T., Ohta S., Morisaki H. (2016). Feasibility and safety of hydrogen gas inhalation for post-cardiac arrest syndrome. Circ. J..

[20] Guan W.J., Wei C.H., Chen A.L., Sun X.C., Guo G.Y., Zou X., Shi J.D., Lai P.Z., Zheng Z.G., Zhong N.S. (2020). Hydrogen/oxygen mixed gas inhalation improves disease severity and dyspnea in patients with Coronavirus disease 2019 in a recent multicenter, open-label clinical trial. J. Thorac. Dis..

[21] Gong Z., Guan J., Ren X., Meng D., Zhang H., Wang B., Yan X. (2016). Protective effect of hydrogen on the lung of sanitation workers exposed to haze. Chin. J. Tuberc. Respir. Dis..

[22] Kajiyama S., Hasegawa G., Asano M., Hosoda H., Fukui M., Nakamura N., Kitawaki J., Imai S., Nakano K., Ohta M. (2008). Supplementation of hydrogen-rich water improves lipid and glucose metabolism in patients with type 2 diabetes or impaired glucose tolerance. Nutr. Res..

[23] Xia C., Liu W., Zeng D., Zhu L., Sun X., Sun X. (2013). Effect of hydrogen-rich water on oxidative stress, liver function, and viral load in patients with chronic hepatitis B. Clin. Trans. Sci..

[24] Song G., Li M., Sang H., Zhang L., Li X., Yao S., Yu Y., Zong C., Xue Y., Qin S. (2013). Hydrogen-rich water decreases serum low-density lipoprotein cholesterol levels and improves high-density lipoprotein function in patients with potential metabolic syndrome. J. Lipid Res..

[25] Saitoh Y., Okayasu H., Xiao L., Harata Y., Miwa N. (2008). Neutral pH hydrogen-enriched electrolyzed water achieves tumor-preferential clonal growth inhibition over normal cells and tumor invasion inhibition concurrently with intracellular oxidant repression. Oncol. Res..

[26] Saitoh Y., Yoshimura Y., Nakano K., Miwa N. (2009). Platinum nanocolloid-supplemented hydrogen-dissolved water inhibits growth of human tongue carcinoma cells preferentially over normal cells. Exp. Oncol..

[27] Asada R., Kageyama K., Tanaka H., Matsui H., Kimura M., Saitoh Y., Miwa N. (2010). Antitumor effects of nano-bubble hydrogen-dissolved water are enhanced by coexistent platinum colloid and the combined hyperthermia with apoptosis-like cell death. Oncol. Rep..

[28] Saitoh Y., Ikeshima M., Kawasaki N., Masumoto A., Miwa N. (2016). Transient generation of hydrogen peroxide is responsible for carcinostatic effects of hydrogen combined with platinum nanocolloid, together with increase intracellular ROS, DNA cleavages, and proportion of G2/M-phase. Free Radic. Res..

[29] Kato S., Saitoh Y., Miwa N. (2021). Carcinostatic effects of alkanoyl ascorbate plus platinum nano-colloid and stabilization of the esterolytically resultant ascorbate by hydrogen. Hum. Cell.

[30] Kagawa A., Katsura K., Mizumoto M., Tagawa Y., Mashiko Y. (2012). Influence of hydrogen discharged from palladium base hydrogen storage alloys on cancer cells. Mater. Sci. Forum.

[31] Runtuwene J., Amitani H., Amitani M., Asakawa A., Cheng K.C., Inui K. (2015). Hydrogen-water enhances 5-fluorouracil-induced inhibition of colon cancer. PeerJ.

[32] Wang D., Wang L., Zhang Y., Zhao Y., Chen G. (2018). Hydrogen gas inhibits lung cancer progression through targeting SMC3. Biomed. Pharmacol..

[33] Zhao P., Jin Z., Chen Q., Yang T., Chen D., Meng J., Lu X., Gu Z., He Q. (2018). Local generation of hydrogen for enhanced photothermal therapy. Nat. Commun..

[34] Liu M.U., Xie F., Zhang Y., Wang T.T., Ma S.N., Zhao P.X., Zhang X., Lebaron T.W., Yan X.L., Ma X.M. (2019). Molecular hydrogen suppresses glioblastoma growth via inducing the glioma stem-like cell differentiation. Stem Cell Res. Ther..

[35] Yang Y., Liu P.Y., Bao W., Chen S.J., Wu F.S., Zhu P.Y. (2020). Hydrogen inhibits endometrial cancer growth via a ROS/NLRP3/Caspase-1/GSDMD-mediated pyroptotic pathway. BMC Cancer.

[36] Zhao L., Zhou C., Zhang J., Gao F., Li B., Chuai Y., Liu C., Cai J. (2011). Hydrogen protects mice from radiation induced thymic lymphoma in BALB/c mice. Int. J. Biol. Sci..

[37] Kawai D., Takaki A., Nakatsuka A., Wada J., Tamaki N., Yasunaka T., Koike K., Tsuzaki R., Matsumoto K., Miyake Y. (2012). Hydrogen-rich water prevents progression of nonalcoholic steatohepatitis and accompanying hepatocarcinogenesis in mice. Hepatology.

[38] Ye J., Li Y., Hamasaki T., Nakamichi N., Komatsu T., Kashiwagi T., Teruya K., Nishikawa R., Osada K., Toh K. (2008). Inhibitory effect of electrolyzed reduced water on tumor angiogenesis. Biol. Pharm. Bull..

[39] Akagi J., Baba H. (2018). Hydrogen gas restores exhausted CD8+ T cells in patients with advanced colorectal cancer to improve prognosis. Oncol. Rep..

[40] Akagi J., Baba H. (2020). Hydrogen gas activates coenzyme Q10 to restore exhausted CD8^+^ T cells, especially PD-1^+^Timterminal CD8^+^ T cells, leading to better nivolumab outcomes in patients with lung cancer. Oncol. Lett..

[41] Chen J.B., Kong X.F., Lv Y.Y., Qin S.C., Sun X.J., Mu F., Lu T.Y., Xu K.C. (2019). “Real world survey” of hydrogen-controlled cancer: A follow-up report of 82 advanced cancer patients. Med. Gas Res..

[42] Chen J.B., Pan Z.B., Du D.M., Qian W., Ma Y.Y., Mu F., Xu K.C. (2019). Hydrogen gas therapy shrinkage of metastatic gallbladder cancer: A case report. World J. Clin. Cases.

[43] Chen J., Mu F., Lu T., Ma Y., Du D., Xu K. (2019). A gallbladder carcinoma patient with pseudo-progressive remission after hydrogen inhalation. Onco Targets Ther..

[44] Chen J., Mu F., Lu T., Du D., Xu K. (2019). Brain metastases completely disappear in non-small cell lung cancer using hydrogen gas inhalation: A case report. Onco Target Ther..

[45] Chen J.B., Kong X.F., Mu F., Lu T.Y., Lu Y.Y., Xu K.C. (2020). Hydrogen therapy can be used to control tumor progression and alleviate the adverse events of medications in patients with advanced non-small cell lung cancer. Med. Gas Res..

[46] Chen J.B., Kong X.F., Qian W., Mu F., Lu T.Y., Lu Y.Y., Xu K.C. (2020). Two weeks of hydrogen inhalation can significantly reverse adaptive and innate immune system senescence patients with advanced non-small cell lung cancer: A self-controlled study. Med. Gas Res..

[47] Compagni A., Christofori G. (2000). Recent advances in research on multistage tumorigenesis. Br. J. Cancer.

[48] Levine A.J., Oren M. (2009). The first 30 years of p53: Growing ever more complex. Nat. Rev. Cancer.

[49] Sigal A., Rotter V. (2000). Oncogenic mutations of the p53 tumor suppressor: The demons of the guardian of the genome. Cancer Res..

[50] Vousden K.H., Lu X. (2002). Live or let die: The cell’s response to p53. Nat. Rev. Cancer.

[51] Kasai H. (1997). Analysis of a form of oxidative DNA damage, 8-hydroxy-2′-deoxyguanosine, as a marker of cellular oxidative stress during carcinogenesis. Mutat. Res..

[52] Toyokuni S., Mori T., Dizdaroglu M. (1994). DNA base modifications in renal chromatin of Wister rats treated with a renal carcinogen, ferric nitrilotriacetate. Int. J. Cancer.

[53] Tsukahara H., Hiraoka M., Kobata R., Hata I., Ohshima Y., Jiang M.Z., Noiri E., Mayumi M. (2000). Increased oxidative stress in rats with chronic nitric oxide depletion: Measurement of urinary 8-hydroxy-2′-deoxyguanosine excretion. Redox Rep..

[54] Floyd R.A. (1990). The role of 8-hydroxyguanine in carcinogenesis. Carcinogenesis.

[55] Hirano S.-i., Ichikawa Y., Sato B., Yamamoto H., Takefuji Y., Satoh F. (2021). Molecular hydrogen as a potential clinically applicable radioprotective agent. Int. J. Mol. Sci..

[56] Harris S.L., Levine A.J. (2005). The p53 pathway: Positive and negative feedback loops. Oncogene.

[57] Hasty P., Christy B.A. (2013). p53 as an intervention target for cancer and aging. Pathobiol. Aging Age Relat. Dis..

[58] Leonhardt H., Cardoso M.C. (2000). DNA methylation, nuclear structure, gene expression and cancer. J. Cell Biochem. Suppl..

[59] U.S. National Library of Medicine https://clinicaltrials.gov/ct2/home.

[60] Hirano S.-i., Ichikawa Y., Sato B., Yamamoto H., Takefuji Y., Satoh F. (2021). Potential therapeutic applications of hydrogen in chronic inflammatory disease: Possible inhibiting role on mitochondrial stress. Int. J. Mol. Sci..

[61] World Cancer Research Fund International (2018). The Third Expert Report. https://www.wcrf.org/sites/default/files/Summary-of-Third-Expert-Report-2018.pdf.

[62] Mantovani A., Allavena P., Sica A., Balkwill F. (2008). Cancer-related inflammation. Nature.

[63] Greider C.W. (2000). Cellular responses to telomere shortening: Cellular senescence as a tumor suppressor mechanism. Harvey Lect..

[64] Lundberg A.S., Hahn W.C., Gupta P., Weinberg R.A. (2000). Genes involved in senescence and immortalization. Curr. Opin. Cell Biol..

[65] Chambers C.R., Ritchie S., Pereira B.A., Timpson P. (2021). Overcoming the senescence-associated secretory phenotype (SASP): A complex mechanism of resistance in the treatment of cancer. Mol. Oncol..

[66] Kamimura N., Ichimiya H., Iuchi K., Ohta S. (2016). Molecular hydrogen stimulates the gene expression of transcriptional coactivator PGC-1α to enhance fatty acid metabolism. NPJ Aging Mech. Dis..

[67] Radiation Chemistry Data Center, Notre Dame Radiation Laboratory (n.d.) (2011). NERL Data. http://kinetics.nist.gov/solution/.

[68] Wood K.C., Gladwin M.T. (2007). The hydrogen highway to reperfusion therapy. Nat. Med..

[69] Hoovis M.L. (1970). Response of endometrial stromal sarcoma to cyclophosphamide. Am. J. Obstet. Gynecol..

[70] Kim R., Nishimoto N., Inoue H., Yoshida K., Toge T. (2000). An analysis of the therapeutic efficacy of protracted infusion of low-dose 5-fluorouracil and cisplatin in advanced gastric cancer. J. Infect. Chemother..

[71] Levine M. (2000). Epirubicin in breast cancer: Present and future. Clin. Breast Cancer.

[72] Sekine I., Saijo N. (2000). Novel combination chemotherapy in the treatment of non-small cancer lung cancer. Expert Opin. Pharmacother..

[73] Hirano S.-i., Agata N., Hara Y., Iguchi H., Shirai M., Tone H., Urakawa N. (1991). Effects of pirarubicin, an antitumor antibiotic, on the cardiovascular system. Cancer Chemother. Pharmacol..

[74] Hirano S.-i., Agata N., Hara Y., Iguchi H., Shirai M., Tone H., Urakawa N. (1991). Pirarubicin-induced endotherium-dependent relaxation in the rat isolated aorta. J. Pharm. Pharmacol..

[75] Hirano S.-i., Agata N., Hara Y., Iguchi H., Shirai M., Tone H., Urakawa N. (1992). A possible mechanism of endothelium-dependent relaxation induced by pirarubicin and carbachol in rat isolated aorta. J. Pharm. Pharmacol..

[76] Hirano S.-i., Agata N., Iguchi H., Tone H. (1995). Effects of pirarubicin in comparison with epirubicin and doxorubicin on the contractile function in rat isolated cardiac muscles. Gen. Pharmacol..

[77] Rochette L., Zeller M., Cottin Y., Vergely C. (2021). Antitumor activity of protons and molecular hydrogen underlying mechanism. Cancer.

[78] Yang Y., Zhu Y., Xi X. (2018). Anti-inflammatory and antitumor action of hydrogen via reactive oxygen species. Oncol. Lett..

[79] Klein E.A., Thompson I.M., Tangen C.M., Growley J.J., Lucia M.S., Goodman P.J., Minasian L.M., Ford L.G., Parnes H.L., Gaziano J.M. (2011). Vitamin E and the risk of prostate cancer. The selenium and vitamin E cancer prevention trial (Select). J. Am. Med. Assoc..

[80] Chandel N.S., Tuveson D.A. (2014). The promise and perils of antioxidants for cancer patients. N. Engl. J. Med..

[81] Sayin V.I., Ibrahim M.X., Larsson E., Nilsson J.A., Lindahl P., Bergo M.O. (2014). Antioxidants accelerate lung progression in mice. Sci. Transl. Med..

[82] DeNicola G.M., Karreth F.A., Humpton T.J., Gopinathan A., Wei C., Frese K., Mangal D., Yu K.H., Yeo C.J., Calhoun E.S. (2011). Oncogene-induced Nrf2 transcription promotes ROS detoxification and tumorigenesis. Nature.

[83] Schafer Z.T., Grassian A.R., Song L., Jiang Z., Gerhart-Hines Z., Irie H.Y., Gao S., Puigserver P., Brugge J.S. (2009). Antioxidant and oncogene rescue of metabolic defects caused by loss of matrix attachment. Nature.

[84] Yang Y., Li B., Liu C., Chuai Y., Lei J., Gao F., Cui J., Sun D., Cheng Y., Zhou C. (2012). Hydrogen-rich saline protects immunocytes from radiation-induced apoptosis. Med. Sci. Monit..

[85] Zhao S., Yang Y., Liu W., Xuan Z., Wu S., Yu S., Mei K., Huang Y., Zhang P., Cai J. (2014). Protective effect of hydrogen-rich saline against radiation-induced immune dysfunction. J. Cell Mol. Med..

[86] Ozeki N., Yamawaki-Ogata A., Narita Y., Mii S., Ushida K., Ito M., Hirano S.-i., Kurokawa R., Ohno K., Usui A. (2019). Hydrogen water alleviates obliterative airway disease in mice. Gen. Thorac. Cardiovasc. Surg..

[87] Liu C., Kurokawa R., Fujino M., Hirano S.-I., Sato B., Li X.K. (2014). Estimation of the hydrogen concentration in rat tissue using an airtight tube following the administration of hydrogen via various routes. Sci. Rep..

[88] Yamamoto R., Homma K., Suzuki S., Sano M., Sasaki J. (2019). Hydrogen gas distribution in organs after inhalation: Real-time monitoring of tissue hydrogen concentration in rat. Sci. Rep..

